# Association between hyperuricemia and glycated hemoglobin in type 2 diabetes at the District Hospital of Dschang

**DOI:** 10.11604/pamj.2021.40.177.30207

**Published:** 2021-11-22

**Authors:** Martial Donkeng, Dieudonné Kuaté, Patrice Nzogang Koudjou, Jean Jacques Noubiap, Jules Roger Kuiate

**Affiliations:** 1 University of Dschang, Dschang, Cameroon,; 2 University of Cape Town, Groote Schuur Hospital, Cape Town, South Africa

**Keywords:** Uric acid, glycated haemoglobin, type 2 diabetes

## Abstract

**Introduction:**

given the difficulty to obtain glycemic control in type 2 diabetes, the exploration of others pathophysiological hypotheses could improve the understanding of this phenomenon. We conducted this study to search for an association between uric acid and uncontrolled diabetes.

**Methods:**

it was a cross-sectional study in a population of type 2 diabetes with normal postprandial and fast blood sugar for the last three months at the District Hospital of Dschang. Chi square test were used to analyzed categorical variables and Pearson correlation coefficient to evaluate the relationship between uric acid level and HbA1c. Means were compared using Kruskal-Walli’s or Student's test.

**Results:**

our study of 80 patients found that, female sex and central obesity were risk factors for hyperuricemia. Prevalence of hyperuricemia and uncontrolled diabetes was respectively 27.5% and 33.8%. The proportion of women with hyperuricemia was significantly higher than that of men (59.1% versus 40.9%, p=0.021) and this inequality of proportion was more marked particularly after 45 years (40% versus 18.8%, p=0.022). Hyperuricemia was not significantly associated to uncontrolled diabetes (OR=2.01 IC (0.73-5.52), p=0.095). Serum uric acid was positively and significantly correlated to glycated hemoglobin (r=0.318, p=0.002) and hyperuricemia was significantly correlated to uncontrolled diabetes (r=0.712, p=0.035). The mean of glycated hemoglobin is abnormal (7.14%) when uric acid level is around 5.75 mg/dl without sex distinction.

**Conclusion:**

these results suggest that the rate of glycated hemoglobin is associated to serum uric acid variations during type 2 diabetes.

## Introduction

Type 2 diabetes (T2D) is steadily increasing worldwide, with 246 million people affected in 2007, 425 million in 2017 and 212.4 million undiagnosed cases [1]. This disease causes significant number of morbidities and these morbidities are mainly due to vascular lesions. These vascular lesions observed in T2D have multifactorial origin; however, poor glycemic control seems to be their root causes [2].

Uric acid is a biochemical entity that progressively arouses the curiosity of researchers because of its potential role in the pathophysiology of T2D complications. Elevation of its plasma concentration is not uncommon during T2D. The prevalence of hyperuricemia during T2D ranges between 16 to 38% according to studies [3-5]. In T2D, hyperuricemia is frequently associated to cardiovascular risk factors such as dyslipidemia, insulin resistance, high blood pressure, central obesity and smoking [6,7]. In addition, hyperuricemia directly increases the risk of cardiovascular diseases in exposed individuals [8].

It is recognized that, hyperuricemia plays a role in the development of metabolic syndrome. During metabolic syndrome, resistance to insulin which is at the center of its pathophysiology could originate from the oxidative stress generated by hyperuricemia due to disorder cause by obesity [9-12]. Given the difficulty to obtain glycemic control in diabetic patients, the verification of new pathophysiological hypotheses could improve the understanding of this phenomenon. The participation of hyperuricemia in the genesis of the metabolic syndrome suggests that it may participate in poor glycemic control during diabetes.

The general objective of this study was to investigate the association between uric acid and glycated haemoglobin (HbA1c) in type 2 diabetic subjects.

## Methods

### Study population

We conducted a cross-sectional study during a period of three months from October to November 2016. The studied population was made up of diabetic patients followed up at the District Hospital of Dschang, a third category hospital of the Menoua Department (West Cameroon). Only followed-up patients with T2D were included in the study. Diabetic patients who did not observe their treatment, those who were hospitalized for the past three months and those who had high fasting blood sugar (up to 130mg/ml) or high postprandial glycaemia (up to 180 mg/ml) during the past three months were excluded. Similarly, patients with gout, under hypouricemic treatment and pregnant women were excluded. Ethic clearance was obtained from the Regional Ethic Committee for Human Health Research of Centre. Patients who gave their oral consent were interviewed. Information was collected on sex, age, age of diabetes, diagnosed complications or associated diseases (hypertension, myocardial infarction, kidney failure, neuropathies, retinopathies, strokes, diabetic food), diet, physical activities and treatment. The waist circumference (WC) was measured by applying the measuring tape at mid-height between the lower edge of the last rib and the upper edge of the iliac crest. Blood pressure was measured in a sitting position after 20 minutes of rest by applying sphygmomanometer at mid-height of the right arm. The glycated hemoglobin result reflects the mean daily blood glucose concentration over the preceding two to three months. Uncontrolled diabetes was defined as a HbA1c greater than 7%. Hyperuricemia was reported for uric acid levels above 6 mg/dl in women and above 7 mg/dl in men. Obesity was considered in participants with waist circumference greater than 88 cm for women and 102 cm for men. Sedentarity was defined as physical inactivity. Hypertension was defined by a systolic blood pressure up to 140 mmHg and a diastolic blood pressure up to 90 mmHg [13]. The sample size was estimated using the Lorentz´s sample size formula. Using prevalence of 4.9% of type 2 diabetic in Africa [2], the minimum required sample size was 72.

### Setting

A blood sample of five milliliters was collected by venipuncture at the bend of the elbow after at least 8 hours of fasting between nine and twelve AM. The glycated haemoglobin is formed by the reversible condensation of the carbonyl group of glucose and the amino group at the N-terminus of the beta chain of hemoglobin A. The assay was performed by ions exchange chromatography on micro column. Uric acid was assayed by the enzymatic colorimetric method. The optical densities were measured using the BioSystem BTS-310 spectrophotometer at a wavelength of 420 nm and 510 nm, respectively.

### Statistical analysis

The data were analyzed with the Epi Info software version 3.5.4 for windows and using the Statistical Package for Social Science (SPSS) version 20.0 for Windows (SPSS, Chicago, Illinois, USA). The categorical variables were analyzed with the Chi square test. The Pearson correlation coefficient was used to evaluate the relationship between uric acid level and HbA1c. Quantitative variables were subjected to analysis of variance (ANOVA). When there existed differences, the means were compared using the Kruskal-Wallis tests. When the differences didn´t exist, they were compared with the student´s test. The confidence interval was set at 95% with a significance threshold of less than 5% (p < 0.05).

## Results

On 114 diabetic patients received for follow-up during the study period, 80 were included in the study. The participants were aged between 29 to 83 years old with a mean age of 61.22±12.27 years. The average age of men was 64.69±10.1 years and it was significantly higher (p=0.001) than that of women (55.74±13.51 years). The sex ratio was 3: 2 with 49 men and 31 women. Obesity was observed in 62.5% of the population. The proportion of obese women was 93.5% and it was significantly higher than that of men 42.5% (n=41) (p=0.000). Most participants (92.5%) were taking oral antidiabetic drugs, 7.5% were taking insulin therapy and none under both. Sedentarity was described in 26.5% of the participant and 45% nibbled between meals.

Hypertension was observed in 60% of the study population. The proportion of men with hypertension 61.3%, was comparable to that of women 59.2% p=0.851. Hypertension was not significantly associated to uncontrolled diabetes OR 0.65 (0.07-8.99), p=0.510. The mean systolic blood pressure of uncontrolled diabetic was 141 ± 14.3 mmHg and the mean of diastolic blood pressure of the same group was 86 ± 8.2 mmHg. The classes of antihypertensive agent used were calcium channel blockers, Angiotensin Converting Enzyme (ACE) inhibitors, thiazide diuretics.

Hyperuricemia was observed in 22 participants, representing an overall prevalence of 27.5%. The mean age in the group of participants with hyperuricemia was comparable to that of the normouricemic group (respectively 60.59 ± 13.40, 1.46 ± 11.93, p=0.778). Women were more affected by hyperuricemia than men (respectively 59.1%, 40.9%; p=0.021). The proportion of women over 45 years old with hyperuricemia was statistically higher than that of men in the same age group (respectively 40%, 18.8%; p=0.022). In the univariate analysis presented in Table 1, female sex and central obesity were risk factors of hyperuricemia. Consumption of purine-rich foods, high blood pressure, insulin therapy, diabetes over 3 years, and thiazide diuretics were not significantly associated to hyperuricemia.

**Table 1:** comparison of clinical parameters between hyperuricemic and normouricemic subjects

Parameters	Hyperuricemia	Normal uric acid	p value
Age (years)	60.59±13.40	61.46±11.93	0.778
Age of diabetes (years)	5.20±5.38	5.01±4.21	0.867
WC (cm)	102.54±6.95	97.58±12.69	0.063
HbA1c (%)	7.58±2.45	6.24±1.53	0.0047

The prevalence of uncontrolled diabetes was 33.8%. In the group of hyperuricemic participant, 45.5% were uncontrolled and the mean of HbA1c of uncontrolled patients was 9.24±2.85% vs 6.19±0.50% in controlled patients. Patients with hyperuricemia had significantly higher HbA1c than normouricemic patients (Table 2). Comparison of the mean of HbA1c between participants with a uric acid level less than 5.75 mg/dl to those with a level greater than 5.75 mg/dl showed abnormal elevation of 1.11% (respectively 6.03%, 7.14%; p=0.008).

**Table 2:** hyperuricemia associated factors

Variable	Hyperuricemia n (%)	Normal uric acid n (%)	OR (CI at 95%)	p-value
Female	13 (41.9%)	18 (58.9%)	3.21 (1.03-10.10)	0.021
Female >45 years	11 (44%)	2 (33.3%)	1.57 (0.24-10.22)	0.500
Urban zone	14 (28.6%)	35 (71.4%)	1.15 (0.37-3.69)	0.498
Diabetes>3 years	10 (20.8%)	38 (79.2%)	0.43 (0.83-6.18)	0.084
Hypertension	9 (37.5%)	15 (62.5%)	3.60 (0.33-183.67)	0.250
Thiazide diuretic	5 (33.3%)	10 (66.7%)	0.62 (0.08-4.77)	0.453
Nibbles	12 (33.3%)	24 (66.7%)	1.70 (0.56-5.16)	0.210
Alcohol	8 (22.2%)	28 (77.8%)	0.61 (0.19-1.91)	0.250
High purine diet	21 (27.3%)	56 (72.7%)	0.75 (0.03-46.33)	0.624
Sedentarity	6 (28.6%)	15 (71.4%)	1.07 (0.28-3.60)	0.553
Obesity	19 (38%)	31 (62%)	5.51 (1.37-31.64)	0.005
Systolic hypertension	15 (36.6%)	26 (63.4%)	2.64 (0.94-7.34)	0.081
Diastolic hypertension	9 (39.1%)	14 (60.9%)	2.18 (0.77-6.16)	0.170

**Figure 1:**
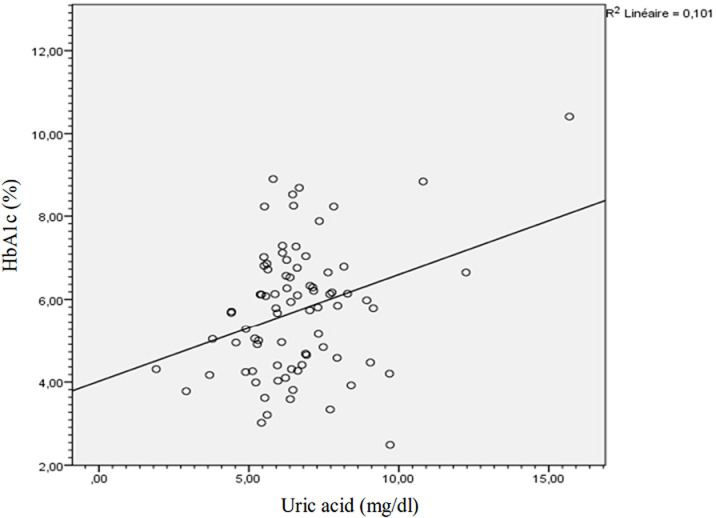
correlation between uric acid and glycated hemoglobin

The analysis of the correlation between clinico-biological parameters and serum uric acid reveals a significant positive and linear correlation between uric acid and glycated hemoglobin (Figure 1). There was a positive and significant correlation between uric acid and HbA1c when HbA1c is greater than 7% (r=0.414, p 0.036) and there was equally the case when HbA1c is lower than 7% (r=0.305, p=0.025). Hyperuricemia was strongly and positively correlated to high HbA1c (r=0.712, p=0.035). Obesity was not significantly associated to uncontrolled diabetes OR 1.68 (0.57-5.26), p=0.214.

## Discussion

The evaluation of the relationship between uric acid and HbA1c in type 2 diabetes is rare in literature. In this study, we discovered that hyperuricemia is common in the group of participants with HbA1c upper than 7%. In fact, 37% of participants with high HbA1c have hyperuricemia. Wang *et al*. equally observed a great prevalence, 30.3% [6]. This group of participants was predominantly obese. In this study, 86.3% of participants with hyperuricemia were obese with a mean waist circumference of 102.5 cm and obesity was seen as a risk factor for hyperuricemia (OR=5.51, p 0.005). The association between obesity that characterizes the metabolic syndrome and hyperuricemia during type 2 diabetes has often been described in others epidemiological studies [3,5,6,14]. One of the common situations between early stage of type 2 diabetes and metabolic syndrome is hyperinsulinism with resistance to insulin [11]. Hyperuricemia could come from this hyperinsulinism by two ways: Insulin stimulates uric acid reabsorption from the proximal convoluted tubule and the lactic acid produced during diabetes reduces the renal tubular secretion of uric acid through competitive inhibition of uric acid on its receptor [15].

During hyperuricemia, diabetes is frequently non-controlled (45.5%) and several modifications of HbA1c relevant to hyperuricemia can be describe here. Hyperuricemia appears as associated factor of uncontrolled diabetes. The mean HbA1c of the participants with hyperuricemia was significantly higher than that of those with normal uric acid levels. But, only less than 50% of participants with hyperuricemia were uncontrolled. These results suggest that participants with hyperuricemia are not always uncontrolled, but when the imbalance exists, the HbA1c level would be very high (9.24 ± 2.85%). There is a significant positive and crescent correlation between uric acid and HbA1c. This observation assumes that the level of uric acid increases with that of HbA1c. The existence of a correlation between uric acid and HbA1c less than 7% and the existence of a strong correlation between hyperuricemia and HbA1c greater than 7% reinforces this idea. Uric acid appears to be associated with HbA1c during type 2 diabetes. But the correlation obtained here is weak and the existence of isolated points on the correlation diagram (Figure 1) could increase it. Exploration of this relationship led us to search for a threshold of uric acid from which HbA1c becomes abnormal. We have observed that HbA1c becomes abnormal for a uric acid level of 5.75 mg/dl without distinction of sex. The description of this relationship then led us to examine the determinants of hyperuricemia during type 2 diabetes.

Uric acid elevation is an event that occurs most often during diabetes. Our study reports a prevalence of 27.5%. This prevalence is greater than that giving by some authors [3]. Hyperuricemia has multiple origin during diabetes. It could come from an excess intake of food and equally from endogenous disturbances. Diet of the respondents were not seen to be risk factors of hyperuricemia. As far as alcohol, which significantly increases the level of uric acid [16]. However, these results must be taken in hindsight; given that the information on the methods of preparing meals, the quantities consumed each time, the rate of consumption of purine-rich foods have not been considered. Looking that diet is not a risk factor of hyperuricemia here, uric acid production could be of endogenous origin. Uric acid could be produced in response to oxidative stress induced by chronic exposure to hyperglycemia that could be attributed here to the proportion of uncontrolled participant, 33.8%. This chronic exposure to hyperglycemia causes glucose self-oxidation and glycation of proteins responsible for production of reactive oxygen species. Uric acid is a powerful reducing agent that is involved in the first line of the reaction to oxidative stress [17].

Associated factors to hyperuricemia were female sex and central obesity. High blood pressure and thiazide diuretic were not associated to hyperuricemia. The proportion of women with hyperuricemia was statistically higher than that of men. After stratifying the population with hyperuricemia by age and sex, the proportion of women over 45 years was statistically higher than that of men. We equally observe that women age above 45 years are more at risk of hyperuricemia than women of less than 45 years. Considering 45 years as menopausal age, this result could let us think that serum uric acid is higher in menopausal women. This result corroborates that of some authors who describe menopause as risk factor of hyperuricemia [6].

**Limitations:** the size of our sample is weak as well as the correlation obtained. Our results could be a characteristic of the study population but these weaknesses make them purely suggestive.

## Conclusion

The existence of an association between uric acid and HbA1c during type 2 diabetes is an issue used in the monitoring of uric acid levels in these patients. These levels could be used as an indicator of glycaemic control, specifically because it is possible to establish uric acid threshold from which HbA1c becomes abnormal. Nevertheless, the analysis of this association should be done in conjunction with all the progression factors of type 2 diabetes in a longitudinal study to verify our observations.

### What is known about this topic

- Elevation of plasma concentration of uric acid is frequent during T2D;
- Hyperuricemia directly increases the risk of cardiovascular disease in exposed individuals and plays a role in the development of metabolic syndrome.

### What this study adds

- There was a significant positive and linear correlation between uric acid and glycated hemoglobin (r=0.318; p=0.002);
- Hyperuricemia was strongly and positively correlated to high HbA1c (r=0.712, p=0.035);
- The level of HbA1c becomes abnormal starting with a uric acid level ≥ 5.75 mg / dl (p=0.008).
